# General anesthesia vs. conscious sedation for endovascular treatment of acute basilar artery occlusion: a secondary analysis of the ATTENTION trial

**DOI:** 10.3389/fneur.2026.1772754

**Published:** 2026-04-02

**Authors:** Bin Han, Raynald, Muhammad Firdaus, Yaxin Wu, Ganghua Feng, Xuehan Liu, Peng Zhang, Pengyu Lu, Yi Liu, Wei Hu, Yaxuan Sun

**Affiliations:** 1 Shanxi Key Laboratory of Brain Disease Control, Department of Neurology, Shanxi Provincial People’s Hospital, Taiyuan, China; 2 Department of Neuroscience, Dharmais Cancer Hospital - National Cancer Center, Jakarta, Indonesia; 3 Clinical Research Unit, Dharmais Cancer Hospital - National Cancer Center, Jakarta, Indonesia; 4 The Fifth Clinical Medical College of Shanxi Medical University, Taiyuan, China; 5 Department of Neurology, The First People's Hospital of Chenzhou, Chenzhou First People's Hospital, Institute of Neuromedicine, Chenzhou, China; 6 Division of Life Sciences and Medicine, Department of Neurology, The First Affiliated Hospital of USTC, University of Science and Technology of China, Hefei, China

**Keywords:** acute basilar artery occlusion, anesthetic management, endovascular treatment, outcomes, safety and efficacy

## Abstract

**Purpose:**

This study aimed to evaluate the safety and efficacy of endovascular treatment (EVT) performed under general anesthesia (GA) versus conscious sedation (CS) in patients with acute basilar artery occlusion (aBAO).

**Methods:**

Patients were stratified into GA and CS group. Propensity score matching (1:1) was performed to balanced baseline characteristics. The primary outcome was a favorable functional outcome, defined as a 90-day modified Rankin Scale (mRS) score of 0–2. Secondary outcomes included 90-day mRS 0–1 and 0–3, National Institutes of Health Stroke Scale (NIHSS) scores at 24 and 72 h, successful recanalization, puncture-to-recanalization time, and safety outcomes, including any intracerebral hemorrhage (ICH) and 90-day mortality.

**Results:**

After propensity score matching, there was no significant between the GA and CS groups (adjusted odds ratio [aOR], 0.97, 95% CI, 0.52–1.84; *p* = 0.935). Rates of successful recanalization and puncture-to-recanalization time were also comparable between groups. Ninety-day mortality did not differ significantly between CS and GA (adjusted risk ratio: 1.16; 95% CI: 0.70 to 1.91; *p* = 0.565).

**Conclusion:**

In patients with aBAO undergoing EVT, CS was associated with clinical and safety outcomes comparable to those observed with GA. However, given the modest sample size and limited statistical power, these findings should be interpreted cautiously.

## Introduction

Acute Basilar Artery Occlusion (aBAO) is a highly critical form of stroke associated with high rates of disability and mortality, reaching up to 80% (1–3). The safety and efficacy of Endovascular Therapy (EVT) for aBAO have long been debated. Recently, two prospective, multicenter randomized controlled trials (RCTs) demonstrated that EVT significantly improves functional outcomes compared with Best Medical Management (BMM) in patient with moderate-to-severe aBAO selected according to specific clinical and imaging criteria (4, 5). In this context, periprocedural anesthetic management may play a critical role in influencing outcome.

Most comparative studies of anesthetic strategies during mechanical thrombectomy have focused on anterior circulation stroke, with heterogeneous results (6–12). Several observational studies in BAO have suggested that non–general anesthesia (non-GA) approaches yield clinical and safety outcomes comparable or even superior to general anesthesia (GA) (13–20). Conversely, a limited number of exploratory RCTs have reported no superiority of non-GA for functional independence and have even suggested lower rates of successful recanalization compared with GA (21–24). More recently, a retrospective analysis from the Endovascular Treatment for Acute Basilar Artery Occlusion (ATTENTION) registry found similar clinical and safety outcomes between patients treated with and without GA (25). However, the non-randomized design limits causal inference, underscoring the need for further RCTs to substantiate these findings.

Accordingly, this subanalysis of the prospective multicenter ATTENTION RCT aimed to compare the safety and efficacy of GA versus CS in patients with aBAO undergoing EVT. Unlike previous ATTENTION registry-based analyses, this study is derived from a prospective multicenter randomized controlled trial dataset (ATTENTION trial). The parent trial provides prospective data collection, standardized inclusion criteria, centralized imaging protocols, and more rigorous follow-up assessment. This strengthens internal validity compared with purely retrospective registry studies.

## Methods

### Patient population

The ATTENTION clinical trial was a prospective, multicenter, randomized, open-label study conducted at 36 medical facilities across China. Eligible patients were randomly assigned in a 2:1 ratio to receive endovascular thrombectomy in plus best medical management (thrombectomy group), or best medical management alone (control group). The trial’s comprehensive protocol, accessible via NEJM.org, received ethical approval from leading institution, as well as all pertinent local ethics committees. Prior to randomization, written informed consent was duly obtained from all participating patients or their authorized legal representatives. This study was reviewed and approved by the First Affiliated Hospital of the University of Science and Technology of China and all relevant local ethics committees. The ID of the approval: 2021KY011 (Ethics approved date: 28th January 2021) and procedures followed were in accordance with institutional guidelines and with the 1964 Helsinki declaration and its later amendments or comparable ethical standards. Subjects or their legally authorized representatives provided written informed consent prior to commencing the study (https://www.clinicaltrials.gov; Unique identifier: NCT04751708, 21st February 2021).

**Figure 1:**
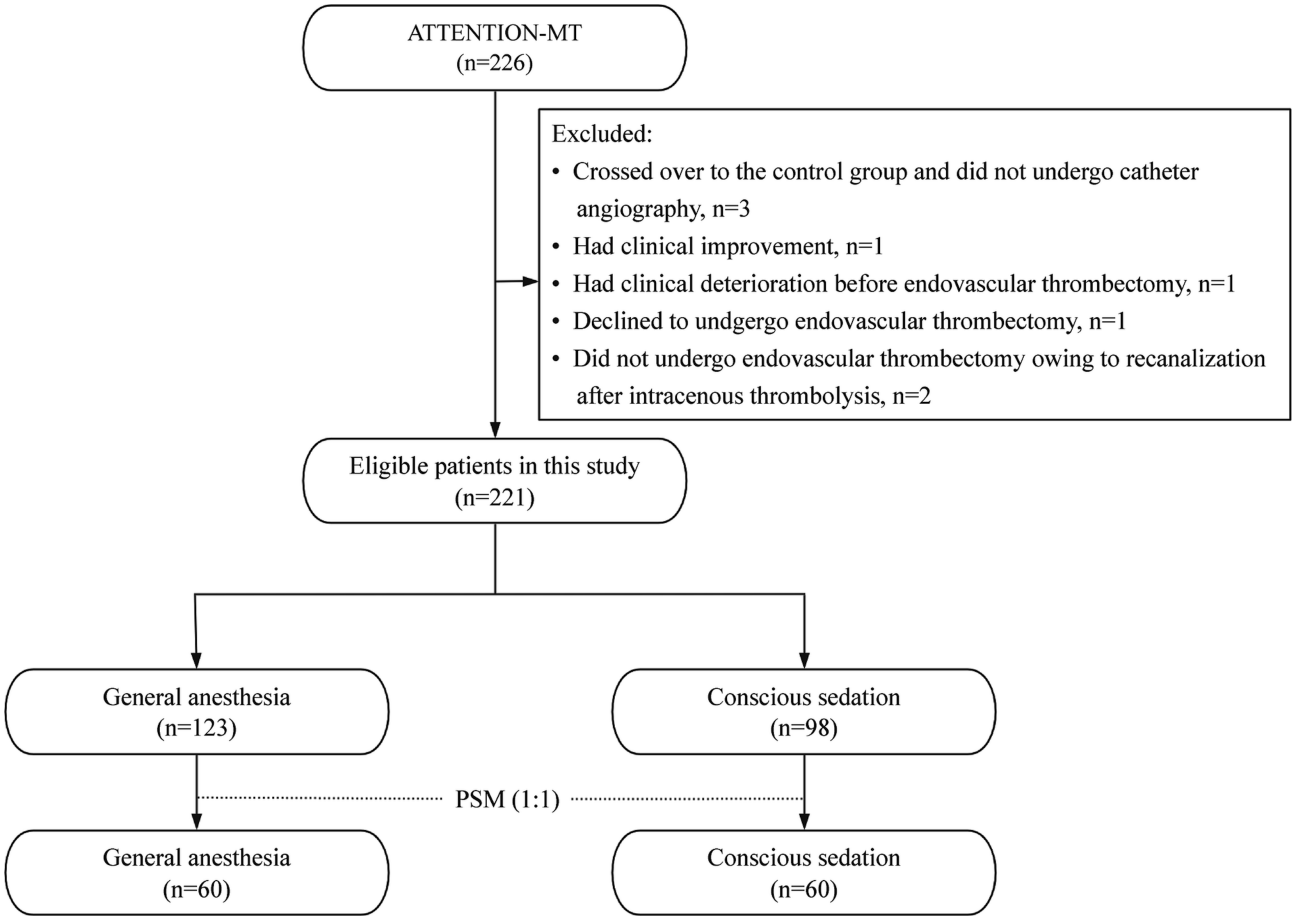
Flow chart of patient selection.

Eligible participants were adults (≥18 years) presenting with moderate-to-severe acute ischemic stroke attributed to basilar artery occlusion (Figure 1). Stroke severity was defined as a National Institutes of Health Stroke Scale (NIHSS) score ≥10 at the time of neuroimaging. Basilar artery occlusion was confirmed by computed tomography angiography (CTA), magnetic resonance angiography (MRA), or digital subtraction angiography (DSA) within 12 h of the estimated symptom onset. The estimated time of occlusion was defined as the onset of sudden basilar artery stroke symptoms, excluding preceding minor prodromal symptoms, and was determined by consensus between two neurologists. In patients with wake-up stroke or unwitnessed onset due to impaired consciousness, the 12-h window was calculated from the time the patient was last known to be well. Imaging to confirm eligibility was primarily performed at participating trial centers, even if prior imaging had been obtained at a referring hospital. Patients with documented basilar artery occlusion at a referring institution but evidence of recanalization on repeat imaging at the trial center were excluded.

Exclusion criteria included a prestroke modified Rankin Scale (mRS) score ≥3 for patients younger than 80 years or ≥1 for those aged 80 years or older; evidence of intracranial hemorrhage on neuroimaging; and a posterior circulation Alberta Stroke Program Early Computed Tomography Score (PC-ASPECTS) < 6 in patients younger than 80 years or <8 in those aged 80 years or older. Among the 507 patients initially enrolled, 114 assigned to the control group were excluded from the present analysis. Of the 226 patients randomized to the thrombectomy group, 3 crossed over to the control group and did not undergo catheter angiography, and 2 were excluded because recanalization was achieved after intravenous thrombolysis and EVT was not performed. The study was approved by the institutional ethics committee and the local ethics committees of all participating centers.

### Baseline data collection

Baseline variables included demographic characteristics, admission National Institutes of Health Stroke Scale (NIHSS) score, blood pressure upon admission, the PC-ASPECTS derived from the initial CT scan. Vascular risk factors were recorded, including hypertension, hypercholesterolemia, diabetes mellitus, atrial fibrillation, smoking, coronary artery disease, heart failure, and prior stroke history. Occlusion location was categorized as proximal, middle, and distal basilar artery, as well as the vertebral artery V4 segment. Stroke etiology was classified as large artery atherosclerosis, cardioembolism, or other/unknown etiology. Workflow time metrics were documented, including onset to randomization, onset to imaging and onset to recanalization times. Procedural details were also collected, including the use of intravenous thrombolysis, intracranial stenting, and balloon angioplasty. All imaging and clinical data were submitted in digital format to a centralized core laboratory. This data encompassed pre-treatment imaging information, which included non-contrast CT scans, MRI scans, and DSA images acquired during EVT, as well as subsequent follow-up assessments.

### Anesthetic management

GA was defined as a state of drug-induced unconsciousness necessitating airway protection, typically achieved by tracheal intubation or placement of a laryngeal mask airway. CS was defined as the administration of systemic sedative agents to maintain patient comfort and cooperation during the procedure without the need for advanced airway protection. The selection of the preferred approach for anesthetic management was left to the discretion of the treatment team. Notably, patients who transitioned from CS to a state of GA were subsequently categorized within the GA group for analysis and evaluation.

### Outcome measures

The primary outcome was the distribution of the modified Rankin Scale (mRS) score at 90 days. Functional outcomes were assessed by board-certified vascular neurologists as part of standard care for all stroke patients across the participating centers. Local investigators received specific guidelines to evaluate the mRS score at 90 days through either structured telephone interviews or in-person evaluations. Secondary outcomes included the proportion of patients achieving mRS scores of 0–1, 0–2, and 0–3 at 90 days, as well as NIHSS scores at 24 h and 3 days after the procedure. Successful reperfusion was assessed based on the post-intervention modified Thrombolysis in Cerebral Infarction (mTICI) score, a scale ranging from grade 0 (indicating no reperfusion) to grade 3 (indicating complete reperfusion) (26). The mTICI grading was assessed based on procedural angiography, with imaging data transferred to the central core laboratory. Reperfusion status was evaluated using the modified Thrombolysis in Cerebral Infarction (mTICI) scale (range, 0 [no reperfusion] to 3 [complete reperfusion]). Angiographic images were centrally adjudicated by the core laboratory. Successful recanalization was defined as an mTICI grade of 2b or 3. Puncture-to-recanalization time was also recorded.

### Statistical analysis

Descriptive statistics were stratified according to anesthetic strategy (GA vs. CS). Continuous variables are presented as mean ± standard deviation (SD) or medians along with their respective interquartile ranges (IQR), while categorical variables were conveyed as frequencies with corresponding percentages. Standardized mean differences were calculated to assess baseline balance between groups. Group comparisons were performed using the Student t-test or the Mann–Whitney U test for continuous variables, while the chi-square test or the Fisher exact test for categorical variables. For the primary outcome, univariate associations were initially evaluated using logistic regression. Adjusted relative risks (RRs) and 95% confidence intervals (CIs) were subsequently estimated using a modified Poisson regression model, adjusting for covariates with *p* < 0.10 in univariate analysis. Secondary outcomes were analyzed using generalized linear models with appropriate link functions (Poisson or normal distribution, as applicable), applying similar adjustment strategies. Effect estimates are reported as relative risks with corresponding 95% CIs. All tests were two-sided, and statistical significance was defined as *p* < 0.05. Potential confounders, including balloon angioplasty, stent angioplasty, and hyperlipidemia, were considered in the analysis. To further reduce selection bias and enhance robustness, propensity score matching was performed using the PSMATCH procedure in SAS (version 9.4; SAS Institute Inc.). A 1:1 nearest-neighbor matching algorithm without replacement was applied. Matching variables included age, sex, baseline NIHSS score, PC-ASPECTS, admission blood pressure, vascular risk factors (hypertension, diabetes mellitus, atrial fibrillation, smoking, coronary artery disease, heart failure, hypercholesterolemia, and prior stroke), occlusion site, stroke etiology, use of intravenous thrombolysis, and procedural characteristics (stenting and balloon angioplasty). All statistical analyses were conducted using SAS version 9.4 (SAS Institute Inc.).

## Results

### Baseline characteristics

A total of 221 patients were included, of whom 123 underwent EVT under general anesthesia (GA) and 98 under conscious sedation (CS). Baseline characteristics are summarized in Table 1. The median age of the patient cohort was approximately 65.9 years, with a notable male preponderance (67%). The median baseline NIHSS score at the onset was 24, with IQR ranging from 15 to 35. The distribution of BAO sites was categorized as proximal basilar (30.9%), middle basilar (27.3%), distal basilar artery (32.7%), and vertebral artery V4 segment (9.1%).

**Table 1:** Baseline characteristics of patients undergoing thrombectomy with general anesthesia versus conscious sedation.

Characteristic	All patients (*n* = 221)	General anesthesia (*n* = 123)	Conscious sedation (*n* = 98)	*p* value
Age (y), mean	65.9 ± 11.1	65.5 ± 11.4	66.3 ± 10.8	0.615†
Male, *n* (%)	148 (67)	87 (70.7)	61 (62.2)	0.183*
NIHSS score, median (IQR)	24 (15, 35)	24 (15, 35)	25 (16, 35)	0.679^#^
pc-ASPECTS, median (IQR)	9.0 (8.0, 10.0)	9.0 (8.0, 10.0)	10.0 (8.0, 10.0)	0.034^#^
Systolic blood pressure, median (IQR), mmHg	149.0 ± 20.3	150.5 ± 20.3	147.0 ± 20.3	0.204^†^
Risk factors, *n* (%)				
Hypertension	158 (71.5)	95 (77.2)	63 (64.3)	0.034^*^
Hypercholesterolemia	58 (26.2)	28 (22.8)	30 (30.6)	0.118^*^
Diabetes mellitus	48 (21.7)	28 (22.8)	20 (20.4)	0.673^*^
Atrial fibrillation	45 (20.4)	21 (17.1)	24 (24.5)	0.174^*^
Smoking	61 (27.6)	33 (26.8)	28 (28.6)	0.773^*^
Coronary artery disease	35 (15.8)	22 (17.9)	13 (13.3)	0.350^*^
Heart failure	8 (3.6)	6 (4.9)	2 (2)	0.306^§^
Stroke history	54 (24.4)	32 (26)	22 (22.4)	0.540^*^
Occlusion site				
Proximal basilar artery	68 (30.9)	42 (34.4)	26 (26.5)	0.353^*^
Middle basilar artery	60 (27.3)	34 (27.9)	26 (26.5)	
Distal basilar artery	72 (32.7)	38 (31.1)	34 (34.7)	
Vertebral artery V4	20 (9.1)	8 (6.6)	12 (12.2)	
Stroke cause, *n* (%)				
Large artery atherosclerosis	106 (48)	64 (52)	42 (42.9)	0.085^*^
Cardioembolism	46 (20.8)	19 (15.4)	27 (27.6)	
Other or unknown etiology	69 (31.2)	40 (32.5)	29 (29.6)	
Operation procedure				
Intravenous thrombolysis, *n* (%)	67 (30.3)	33 (26.8)	34 (34.7)	0.206^*^
Stenting, *n* (%)	99 (44.8)	61 (49.6)	38 (38.8)	0.108^*^
Balloon angioplasty, *n* (%)	52 (23.5)	38 (30.9)	14 (14.3)	0.004^*^
Onset-to-randomization, median (IQR), min	5.2 (3.7, 7.2)	5.2 (3.7, 7.2)	5.0 (3.3, 7.2)	0.623^#^
Onset-to-puncture, median (IQR), min	5.6 (3.5, 7.5)	5.6 (3.6, 7.8)	5.5 (3.5, 7.2)	0.412^#^
Time-to-treatment (≤6/>6 h), *n* (%)				
0–6 h	127 (57.5)	64 (52)	63 (64.3)	0.067^*^
>6 h	94 (42.5)	59 (48)	35 (35.7)	

^†^Using the *t*-test; ^*^Using the $\chi^{2}$ test; ^#^Using the Wilcoxon rank-sum test; ^§^Using Fisher exact test.

Values are numbers with percentages in parentheses, unless indicated otherwise; IQR, interquartile range.

NIHSS, National Institutes of Health Stroke Scale; pc-ASPECTS, posterior circulation Alberta stroke program early CT score; TOAST, Trial of ORG 10172 in Acute Stroke Treatment.

The median PC-ASPECTS on admission was lower in GA group than CS group (9 vs. 10). Hypertension was more prevalent in the GA group than CS group (77.2% vs. 64.3%). Balloon angioplasty was performed more frequently in the GA group than CS group (30.9% vs. 14.3%). No significant between-group differences were observed in age, gender, NIHSS score, blood pressure upon admission, and various risk factors (hypercholesterolemia, diabetes mellitus, atrial fibrillation, smoking, coronary artery disease, heart failure, and prior stroke), occlusion site, stroke etiology, use of intravenous thrombolysis, or workflow time metrics (including onset-to-randomization and onset-to-puncture times).

### Outcomes

**Figure 2:**
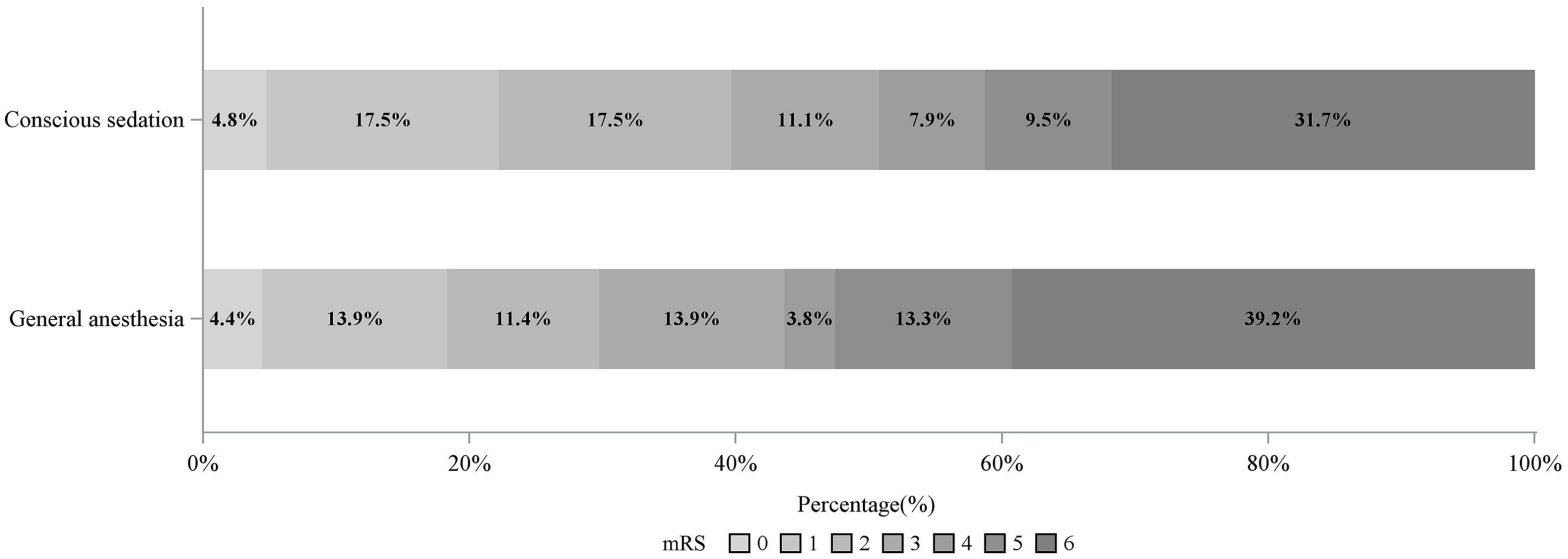
The shift on the 90-d modified Rankin Scale (mRS) score of general anesthesia vs. conscious sedation.

Adjusted analyses are summarized in Table 2. The distribution of 90-day mRS scores with respect to the different anesthetic modality is represented in Figure 2. In the GA group, the 90-day median mRS score was recorded as 5, with an IQR ranging from 2 to 6, while the CS group displayed a three-month median mRS score of 4 (IQR, 2–6). The primary analysis of the primary outcome unveiled no statistically significant difference between the GA and CS groups (adjusted odds ratio [aOR], 0.97; 95% CI, 0.52–1.84; *p* = 0.935). Likewise, the proportion of patients achieving good functional outcomes exhibited no significant difference between the GA and CS groups (aOR: 0.96; 95% CI: 0.60 to 1.52; *p* = 0.851).

**Table 2:** Outcome measures of patients undergoing thrombectomy with general anesthesia versus conscious sedation.

Outcome variables	General anesthesia (*n* = 124)	Conscious sedation (*n* = 99)	Unadjusted analysis		Adjusted model 1^a^		Adjusted model 2^b^	
			Effect size (95% CI)	*P*-value	Effect size (95% CI)	*P*-value	Effect size (95% CI)	*P*-value
Primary outcome								
mRS at 90 d, median (IQR)^c^	5 (2, 6)	4 (2, 6)	0.89 (0.55, 1.42)	0.616	0.89 (0.54, 1.45)	0.633	0.97 (0.52, 1.84)	0.935
Secondary outcomes								
mRS 0–1 at 90 d, *n* (%)^d^	25 (20)	18 (18)	1.11 (0.64, 1.91)	0.716	1.10 (0.64, 1.87)	0.733	1.36 (0.68, 2.72)	0.379
mRS 0–2 at 90 d, *n* (%)^d^	38 (31)	34 (35)	0.89 (0.61, 1.30)	0.549	0.91 (0.62, 1.32)	0.611	0.96 (0.60, 1.52)	0.851
mRS 0–3 at 90 d, *n* (%)^d^	56 (46)	45 (46)	0.99 (0.74, 1.32)	0.954	1.00 (0.75, 1.34)	0.989	1.00 (0.69, 1.45)	1.000
NIHSS score at 24 h, median (IQR)^e^	24 (9, 37)	19 (6, 35)	3.53 (−0.20, 7.26)	0.064	3.23 (−0.58, 7.03)	0.096	5.37 (0.26, 10.47)	0.040
NIHSS score at 72 h, median (IQR)^e^	17 (6, 37)	13 (3, 35)	2.89 (−1.42, 7.19)	0.188	2.81 (−1.59, 7.21)	0.210	3.40 (−2.32, 9.13)	0.242
Successful recanalization at final angiogram (mTICI ≥2b), *n* (%)^d^	114 (93)	92 (94)	0.99 (0.92, 1.06)	0.723	0.98 (0.91, 1.06)	0.650	1.00 (0.91, 1.10)	>0.999
Puncture to recanalization (IQR)^e^	1.42 (0.87, 1.97)	1.09 (0.69, 1.60)	0.35 (0.12, 0.58)	0.003	0.31 (0.07, 0.54)	0.010	0.26 (−0.01, 0.52)	0.061
Safety outcomes								
Any ICH within 24 h, *n* (%)^d^	21 (17)	10 (10)	1.67 (0.83, 3.39)	0.152	1.52 (0.74, 3.15)	0.256	1.80 (0.64, 5.06)	0.265
Death within 90 d, *n* (%)^d^	49 (40)	33 (34)	1.18 (0.83, 1.68)	0.350	1.24 (0.87, 1.77)	0.243	1.16 (0.70, 1.91)	0.565

a Adjusted for the baseline variables with a significant difference of p < 0.05 between both groups, including baseline pc-ASPECTS, hypertension, Balloon, Stroke cause, Time-to-treatment.

b We included pc-ASPECTS, hypertension, Balloon, Stroke cause, Time-to-treatment as potential confounders conducted propensity score matching. Adjusted model 2 was analysis for propensity score matched patients.

c The common OR values were calculated using an ordinal logistic regression model and indicated the odds of improvement of 1 point on the mRS at 90 days.

d The relative risk values were estimation by modified Poisson regression with robust error variance.

e The β-coefficients were calculated using a generalized linear model.

CI, confidence interval; ICH, intracranial hemorrhage; IQR, interquartile range; mRS, modified Rankin Scale; mTICI, modified Thrombolysis in Cerebral Infarction.

At 24 h, the median NIHSS score was higher in the GA group (24 [IQR, 9–37]) than in the CS group (19 [IQR, 6–35]), and this difference remained significant after full adjustment (β = 5.37 [95% CI, 0.26–10.47]; *p* = 0.040). However, the difference in NIHSS scores at 72 h was no longer statistically significant after adjustment (β = 3.40 [95% CI, −2.32–9.13]; *p* = 0.242).

Successful recanalization was achieved in 93% of patients in the GA group and 94% in the CS group, with no significant difference after adjustment (adjusted RR, 1.00 [95% CI, 0.91–1.10]; *p* > 0.999). Regarding procedural efficiency, puncture-to-recanalization time was longer in the GA group (median 1.42 h [IQR, 0.87–1.97]) compared with the CS group (1.09 h [IQR, 0.69–1.60]). In unadjusted and partially adjusted analyses, GA was associated with a significantly longer procedural time (unadjusted β = 0.35 [95% CI, 0.12–0.58]; *p* = 0.003; adjusted model 1 β = 0.31 [95% CI, 0.07–0.54]; *p* = 0.010). After propensity score matching, the association was attenuated and did not reach statistical significance (β = 0.26 [95% CI, −0.01–0.52]; *p* = 0.061), although a numerical trend toward longer procedural duration in the GA group persisted.

**Figure 3:**
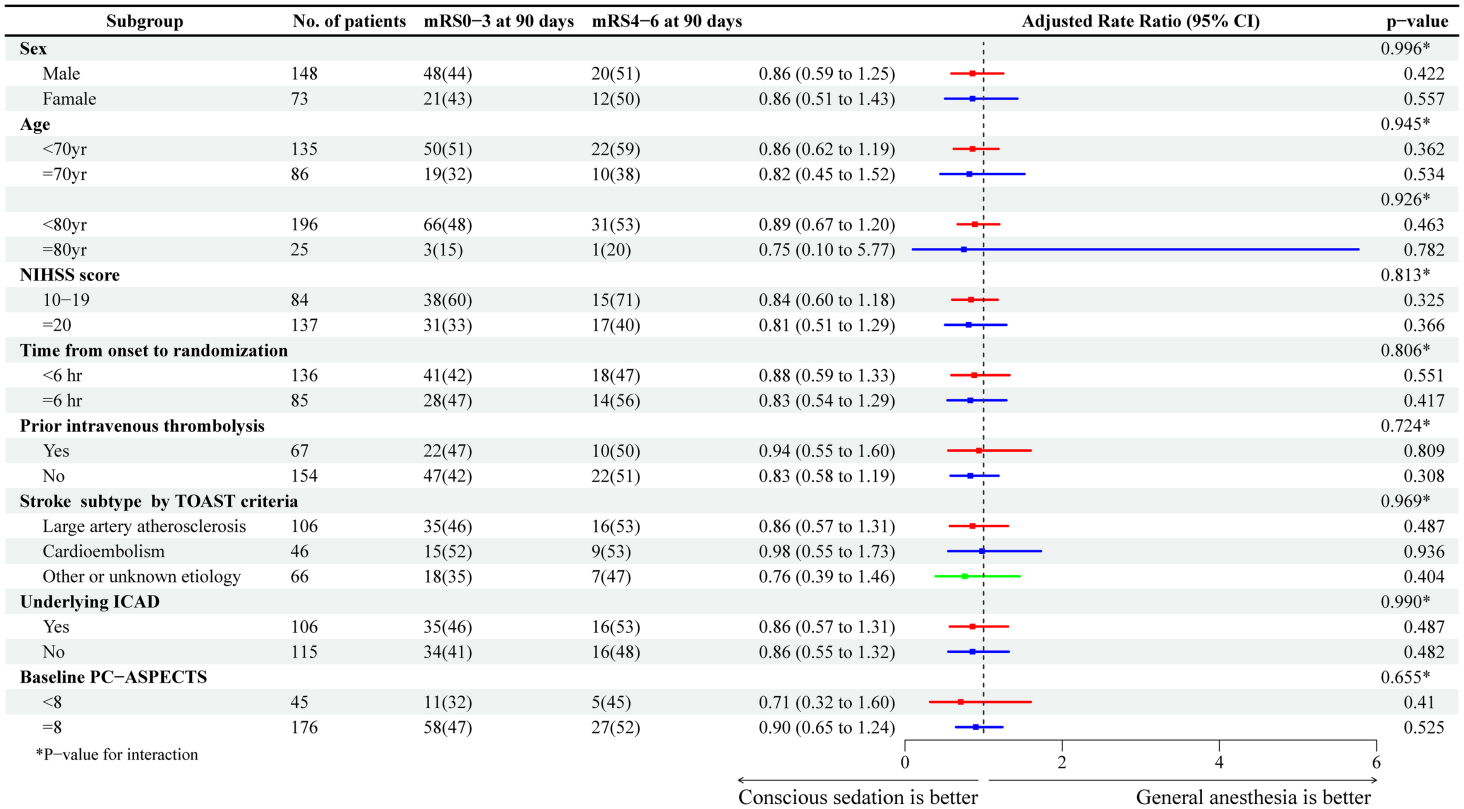
Treatment effects on the primary outcome according to exploratory subgroups.

The 90-day mortality rate was comparable between patients who received CS and those who received GA (adjusted risk ratio: 1.16; 95% CI: 0.70 to 1.91; *p* = 0.565). The likelihood of any ICH within 24 h following the procedure was 17% in patients undergoing GA and 10% in those undergoing CS. Nonetheless, no significant differences emerged between the groups (adjusted risk ratio: 1.80; 95% CI: 0.64 to 5.06; *p* = 0.265). The relationship between the distribution of 90-day mRS scores and the method of anesthetic management was consistent across various subgroups, with no significant interactions observed, as depicted in Figure 3.

## Discussion

In this post-hoc analysis of the ATTENTION trial, anesthetic modality was not associated with functional outcomes at 90-day in patients with aBAO undergoing EVT. CS demonstrated safety outcomes comparable to those of GA.

In anterior circulation acute ischemic stroke, several retrospective studies have suggested that GA may be associated with worse functional outcomes compared with non-GA approaches (6, 8, 27–30). Conversely, other RCTs and retrospective analyses have reported either comparable or superior outcomes with GA (21–24). Evidence in posterior circulation stroke remains limited. Only a few observational studies have addressed this issue, with inconsistent findings, some favoring non-GA strategies (13, 16), while others have reported no significant differences between GA and non-GA (14, 15, 17).

Our findings are consistent with those from the BASILAR and ETIS registries and the CANVAS II trial, which also reported no significant association between anesthetic strategy and functional outcomes in BAO (14, 15, 31). However, noteworthy differences were observed in 24-h NIHSS scores, which were significantly higher in the GA group compared to the non-GA group. GA offers advantages such as airway protection, improved patient immobility, and controlled ventilation. However, its disadvantages primarily involve perioperative hypotension, delayed treatment onset, and the potential for lung infections related to endotracheal tube. These factors could potentially contribute to higher early 24-h NIHSS scores observed in the general anesthesia group compared to the non-GA group, especially for posterior circulation stroke which primarily affects the brainstem.

CS allows maintenance of spontaneous breathing and neurological responsiveness, enabling real-time assessment during the procedure and potentially more stable peri-procedural hemodynamics. Observational data from major thrombectomy trials suggest that a substantial proportion of patients can be safely managed without GA. In ESCAPE and REVASCAT trial, RCTs evaluating EVT in anterior circulation large-vessel occlusion within 12 and 8 h of symptom onset, respectively, approximately 90.9 and 93% of cases utilized non-GA, respectively (32, 33). In the SWIFT PRIME study, another RCT of anterior circulation large-vessel occlusion treated within 6 h, non-GA was adopted in around 63% of cases, demonstrating no adverse impact on postoperative neurological prognosis (34). Similar findings were observed in the THRACE study, an RCT of intravenous thrombolysis plus thrombectomy versus thrombolysis alone in anterior circulation stroke within 4.5 h, where approximately 63% of cases underwent non-GA without affecting postoperative prognosis or reduce recanalization rate (35). CS could be considered as a first-line approach for appropriate and cooperative patients, while acknowledging that stroke severity, procedural complexity, and other clinical factors may necessitate the use of GA. Nevertheless, stroke severity, airway compromise, agitation, or procedural complexity, particularly in cases requiring balloon angioplasty or intracranial stenting, which are common in large-artery atherosclerotic stroke, may necessitate GA. In real-world settings, conversion from CS to GA may be required as a rescue strategy.

In our cohort, the incidence of ICH within 24 h was numerically higher in patients undergoing GA than in those receiving CS (18% vs. 3%), although this difference did not reach statistical significance. This finding should be interpreted cautiously. Anesthetic strategy was not randomized but determined by operator judgment, stroke severity, airway protection requirements, procedural complexity, and institutional practice patterns. Such nonrandom allocation inherently introduces selection bias.

Patients selected for GA were more likely to present with severe neurological deficits, impaired consciousness, agitation, compromised airway reflexes due to posterior circulation involvement, or anticipated technically challenging procedures. Each of these factors is independently associated with worse outcomes and a higher risk of hemorrhagic transformation. Thus, the higher observed rate of ICH in the GA group may reflect greater baseline risk rather than a direct causal effect of anesthetic modality.

This selection mechanism may also explain the numerically longer puncture-to-recanalization time observed in the GA group. Induction of anesthesia, airway management, patient positioning, and hemodynamic stabilization can introduce procedural delays. Moreover, patients requiring GA may have had more complex vascular anatomy, tandem lesions, severe intracranial atherosclerosis, or a greater need for adjunctive interventions such as balloon angioplasty or stenting, factors that can prolong procedure time and potentially increase the risk of hemorrhagic complications. Importantly, detailed anesthesia-related variables, including intraoperative blood pressure variability, duration of hypotension, depth of anesthesia, choice and dosage of anesthetic agents, ventilation parameters, end-tidal CO₂ levels, and use of vasoactive medications, were not systematically collected. These factors may substantially influence cerebral perfusion, collateral circulation, blood–brain barrier integrity, and ultimately the risks of ICH and poor functional outcome. Although multivariable adjustment and propensity score matching were performed to account for measured confounders, residual confounding cannot be excluded.

This study has several limitations. First, although derived from a prospective, multicenter randomized trial dataset, this analysis was *post hoc* and involved a relatively modest sample size, limiting statistical power. Second, detailed intraoperative hemodynamic and ventilation data were unavailable, introducing potential residual confounding. Third, anesthesia-specific details, including airway device type, induction and maintenance agents, depth of anesthesia monitoring, and sedative regimens, were not recorded. Fourth, anesthetic strategy was not randomized but determined by local practice and operator discretion, introducing potential selection bias and center-level variability. Although patients converted from CS to GA were analyzed in the GA group according to protocol, the reasons and timing of conversion were not systematically captured. Finally, the study population consisted exclusively of Chinese patients, which may limit generalizability to other populations with different stroke mechanisms and anesthetic practices. Prospective randomized trials specifically designed to compare anesthetic strategies in posterior circulation stroke are needed.

## Conclusion

In this subgroup analysis, CS showed comparability to GA concerning both functional and safety outcomes among aBAO patients undergoing EVT. Nonetheless, the relatively small sample size, the lack of peri-procedural anesthetic and physiological variables, and limited statistical power restrict definitive conclusions. Further large-scale RCTs are warranted to establish the most appropriate anesthesia strategy for EVT in this setting.

## Data Availability

The original contributions presented in the study are included in the article/supplementary material, further inquiries can be directed to the corresponding authors.
